# A putative siderophore-interacting protein from the marine bacterium *Shewanella frigidimarina* NCIMB 400: cloning, expression, purification, crystallization and X-ray diffraction analysis

**DOI:** 10.1107/S2053230X16011419

**Published:** 2016-08-09

**Authors:** Inês B. Trindade, Bruno M. Fonseca, Pedro M. Matias, Ricardo O. Louro, Elin Moe

**Affiliations:** a Instituto de Tecnologia Química e Biológica António Xavier, Universidade Nova de Lisboa, Avenida da República (EAN), 2780-157 Oeiras, Portugal; b Instituto de Biologia Experimental e Tecnológica (iBET), Apartado 12, 2780-901 Oeiras, Portugal

**Keywords:** siderophore-interacting protein, *Shewanella frigidimarina*, crystallographic analysis

## Abstract

Siderophore-binding proteins (SIPs) perform a key role in iron acquisition in multiple organisms. In the genome of the marine bacterium *Shewanella frigidimarina* NCIMB 400, the gene tagged as SFRI_RS12295 encodes a protein from this family. Here, the cloning, expression, purification and crystallization of this protein are reported, together with its preliminary X-ray crystallographic analysis to 1.35 Å resolution. The SIP crystals belonged to the monoclinic space group *P*2_1_, with unit-cell parameters *a* = 48.04, *b* = 78.31, *c* = 67.71 Å, α = 90, β = 99.94, γ = 90°, and are predicted to contain two molecules per asymmetric unit. Structure determination by molecular replacement and the use of previously determined ∼2 Å resolution SIP structures with ∼30% sequence identity as templates are ongoing.

## Abstract 2

The gene encoding a putative siderophore-interacting protein from the marine bacterium *S. frigidimarina* was successfully cloned, followed by expression and purification of the gene product. Optimized crystals diffracted to 1.35 Å resolution and preliminary crystallographic analysis is promising with respect to structure determination and increased insight into the poorly understood molecular mechanisms underlying iron acquisition.

## 1. Introduction   

The Great Oxygenation Event (GOE) was marked by a profound transition in ocean chemistry, where the previously readily available iron became a limiting nutrient worth battling for (Holland, 2006 ▸). In an oxygen-rich atmosphere, two redox states of iron are accessible to biology: an oxidized, insoluble ferric state, iron(III), and a highly reactive reduced ferrous state, iron(II). The reversibility of this redox pair, iron(II)/iron(III), plays a crucial role in several metabolic processes including the electron-transport chain, photosynthesis, oxidative phosphorylation and the tricarboxylic acid cycle (Miethke & Marahiel, 2007 ▸).

To circumvent low iron bioavailability, organisms have found diverse strategies for importing and utilizing iron, including direct extracellular reduction (Deneer *et al.*, 1995 ▸), the acquisition of iron-bound or haem proteins using specific receptors (Wandersman & Delepelaire, 2004 ▸) and the synthesis and extracellular release of small molecules with high affinity for ferric iron named siderophores (Neilands, 1981 ▸).

These iron siderophores are strong ferric chelators and can be used in various applications, including the cultivation of uncultured microorganisms, as ecofriendly substitutes for pesticides, as enhancers in the bioremediation of heavy metals, in iron-overload therapy, in cancer therapy and as Trojan horse antibiotics (Saha *et al.*, 2016 ▸).

Given their vast applications, considerable efforts have been made in research to understand the mechanistic details behind siderophore synthesis, transport and regulation (Miethke *et al.*, 2011 ▸; Schalk & Guillon, 2013 ▸; Crosa & Walsh, 2002 ▸). At the molecular level, one of the least explored aspects of siderophore use is the release of the imported iron from ferric siderophore complexes. In order to be utilized, iron needs to be released from these high-affinity complexes. Two strategies have been reported so far. One is the release of iron through hydrolytic cleavage of the siderophore complex by esterases (Brickman & McIntosh, 1992 ▸). The other is single-electron reduction, also known as siderophore recycling, which is either mediated by unspecific endogenous reducing agents (Brickman & McIntosh, 1992 ▸) or by specific sidero­phore ferric reductases (Miethke *et al.*, 2011 ▸).

In 2015, Li and coworkers suggested that two families of specific siderophore reductases exist. One is the FAD-containing siderophore-interacting protein (SIP) family and the other is the ferric siderophore reductase (FSR) protein family, which contains an iron–sulfur cluster as a redox centre (Li *et al.*, 2015 ▸). Several members of the SIP family have been characterized [for example, ViuB from *Vibrio cholerae* (Butterton & Calderwood, 1994 ▸), IrtA from *Mycobacterium tuberculosis* (Ryndak *et al.*, 2010 ▸), YqjH from *Escherichia coli* (Miethke *et al.*, 2011 ▸) and FscN from *Thermobifida fusca* (Li *et al.*, 2015 ▸)]. Presently, catalytic mechanisms have been proposed for the SIP family, namely for its members YqjH and FscN. Both of these proteins indicate a preference for NADPH as a reducing agent, although YqjH is able to reduce various iron chelates, whereas FscN is only able to reduce the endogenous siderophore fuscachelin (Miethke *et al.*, 2011 ▸; Li *et al.*, 2015 ▸). Two structures of SIPs have been deposited in the PDB: the 1.89 Å resolution structure of FscN from the Gram-positive *T. fusca* (PDB entry 4yhb; Li *et al.*, 2015 ▸) and the 2.2 Å resolution structure of a SIP from the Gram-negative bacterium *Shewanella putrefaciens* (PDB entry 2gpj; Joint Center for Structural Genomics, unpublished work). Here, we report the cloning, expression, purification and crystallization of a SIP from the marine bacterium *S. frigidimarina* NCIMB 400. Further knowledge of the mechanistic and structural details of this family of proteins, namely with respect to the ligand-binding pockets, may provide new strategies for controlling the performance of siderophore recycling and utilization. This knowledge is relevant for the development of new drugs that inhibit the growth and virulence of pathogens and also for promoting a more efficient use of siderophores in bioremediation *via* the protein engineering of SIPs.

## 2. Materials and methods   

### 2.1. Macromolecule production   

The SFRI_RS12295 gene fragment was amplified *via* polymerase chain reaction (PCR) from the genomic DNA of *S. frigidimarina* NCIMB 400 using the primers listed in Table 1 ▸. The PCR product was ligated into the expression vector pETBlue-1 (Novagen) and transformed into competent *E. coli* Tuner (DE3) pLacI cells for expression. Protein overexpression was achieved using an autoinduction method (Blommel *et al.*, 2007 ▸; Studier, 2005 ▸). The cells were first grown overnight in Luria–Bertani (LB) medium supplemented with 35 mg l^−1^ chloramphenicol and 100 mg l^−1^ ampicillin at 30°C and 170 rev min^−1^. 2% of this culture served as the inoculum. The auto-induction medium was made by adding 50 ml 20× NPS stock solution [1 *M* Na_2_HPO_4_, 1 *M* KH_2_PO_4_, 500 m*M* (NH_4_)_2_SO_4_] and 20 ml 50× 5052 stock solution consisting of 25%(*w*/*v*) glycerol, 2.5%(*w*/*v*) glucose, 10%(*w*/*v*) α-lactose monohydrate to 1 l LB medium supplemented with 1 m*M* MgSO_4_, 35 mg l^−1^ chloramphenicol and 100 mg l^−1^ ampicillin (Blommel *et al.*, 2007 ▸; Studier, 2005 ▸). The cells were allowed to grow continuously for 30 h at 30°C and 170 rev min^−1^.

Cells were harvested by centrifugation for 10 min at 11 305*g* and were then cooled to −80°C. The cells were later defrosted and resuspended in 20 m*M* Tris–HCl buffer pH 7.6 with a protease-inhibitor cocktail (Roche) and DNase I (Sigma) prior to a three-pass cell disruption at 6.9 MPa using a French press. The lysate was centrifuged at 11 305*g* to remove undisrupted cells and then ultracentrifuged at 204 709*g* for 90 min at 4°C to remove cell membranes and debris. The supernatant was dialyzed overnight against 20 m*M* Tris–HCl buffer pH 7.6 and concentrated using an Amicon Ultra Centrifugal Filter (Millipore) with a 10 kDa cutoff. The SIP was purified from the supernatant using two ion-exchange chromatography columns: a Q Sepharose Fast Flow column (GE Healthcare) followed by an SP Sepharose Fast Flow column (GE Healthcare). Both columns had previously been equilibrated with 20 m*M* Tris–HCl buffer pH 7.6 and were used with a stepwise elution method. The fraction containing the SIP eluted from the Q Sepharose column at 150 m*M* NaCl. This fraction was dialyzed against 20 m*M* Tris–HCl buffer pH 7.6, concentrated and loaded onto the SP Sepharose column. The fraction containing the SIP was eluted at 100 m*M* NaCl.

Eluted fractions were analysed by SDS–PAGE with BlueSafe staining (NZYTech) and UV–visible spectroscopy to select fractions containing the purified SIP.

### 2.2. Crystallization   

Purified protein (30 mg ml^−1^) in 20 m*M* Tris–HCl pH 7.6, 150 m*M* NaCl was diluted to 10 mg ml^−1^ with 20 m*M* Tris–HCl pH 7.6 (final salt concentration 50 m*M* NaCl) and used for crystallization experiments on a Honeybee Cartesian crystallization robot (Genomic Systems) with MDL3 plates (100 nl protein solution and 40 µl reservoir solution) and the JCSG-*plus* crystallization screen (Molecular Dimensions). Yellow crystals appeared in 12 out of 96 crystallization conditions. The most promising crystals were found in well A5 with the following reservoir solution: 20% PEG 3350, 0.2 *M* magnesium formate dihydrate. In an attempt to improve the crystal size, hanging-drop optimization experiments were performed by hand, varying the concentration of PEG 3350 from 18 to 22%(*w*/*v*) and of magnesium formate dihydrate from 0.18 to 0.22 *M*. Crystals appeared in all conditions after 1 d and were fully grown after 3 d. In order to confirm that the crystals obtained consist of the SIP, crystals were collected from some of the drops, washed twice in reservoir solution and analysed by SDS–PAGE. The crystallization conditions for the crystal used for data collection are described in Table 2 ▸.

### 2.3. Data collection and processing   

Crystals from several drops were briefly soaked in 22%(*w*/*v*) PEG 3350, 0.2 *M* magnesium formate dihydrate before being flash-cooled in liquid nitrogen or were cooled directly from the crystallization drop in cases where the PEG 3350 concentration was 22%. The best diffracting crystal grew in 22% PEG 3350, 0.22 *M* magnesium formate dehydrate, and data to 1.35 Å resolution were collected on beamline I04 at Diamond Light Source (DLS), UK. A total of 1800 images were collected using 0.1° oscillation width and the data were autoprocessed by *xia*2 (Winter, 2010 ▸), which makes use of *XDS* (Kabsch, 2010 ▸) and the *CCP*4 suite (Winn *et al.*, 2011 ▸) for integration and truncation of the data. The data-collection and processing statistics are listed in Table 3 ▸.

## 3. Results and discussion   

**Figure 1:**
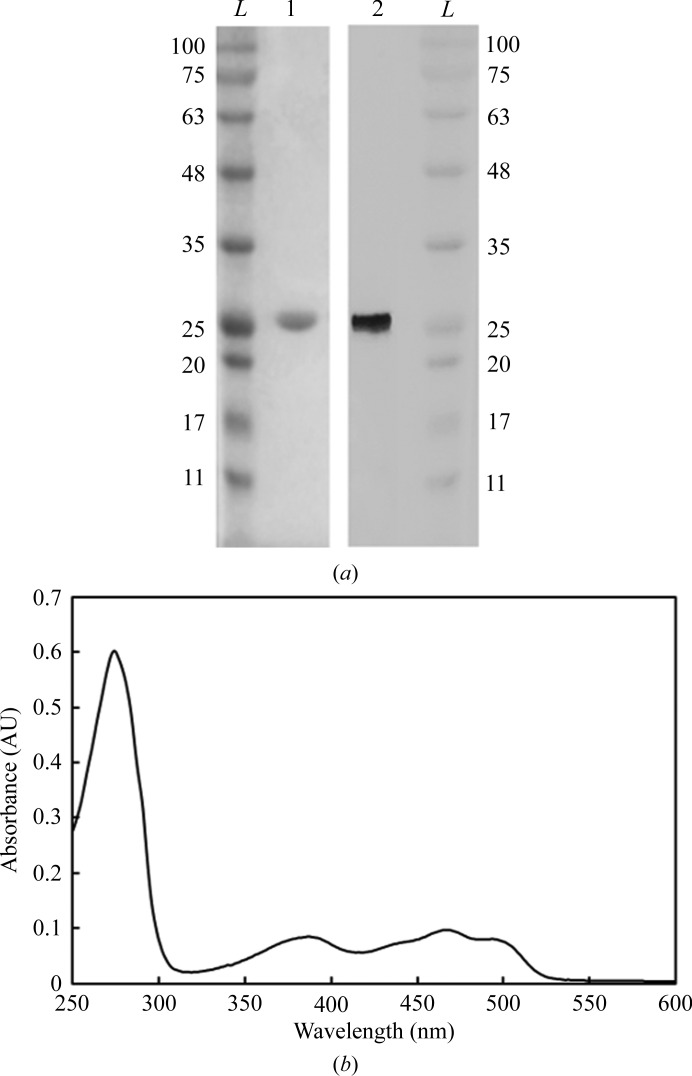
(*a*) 12% SDS–PAGE gels. Lanes 1 and 2 correspond to the purified SIP fraction before crystallization and dissolved multiple crystals of the SIP, respectively. Lane *L* corresponds to the protein ladder (labelled in kDa). (*b*) UV–visible profile of the purified protein. The UV–visible spectrum was measured in a Shimadzu UV-1800 UV–Vis spectrophotometer using a fast scan rate.

The SIP was purified to apparent purity (>95%) by ion-exchange chromatography as described in §[Sec sec2]2. The purified protein appeared yellow and migrated as a single band at ∼30 kDa on a 12% SDS–PAGE gel, as expected from theoretical calculations (Fig. 1 ▸ *a*). Yellow-coloured crystals were dissolved and migrated as a 30 kDa protein on an SDS–PAGE gel, as observed for the purified protein (Fig. 1 ▸ *a*), and confirmed that the crystals consist of the SIP. The purified protein was also analysed by UV–visible spectroscopy and showed typical spectral features of an oxidized flavoprotein in the UV–visible region (Fig. 1 ▸ *b*), with absorption peaks at 387 and 470 nm.

**Figure 2:**
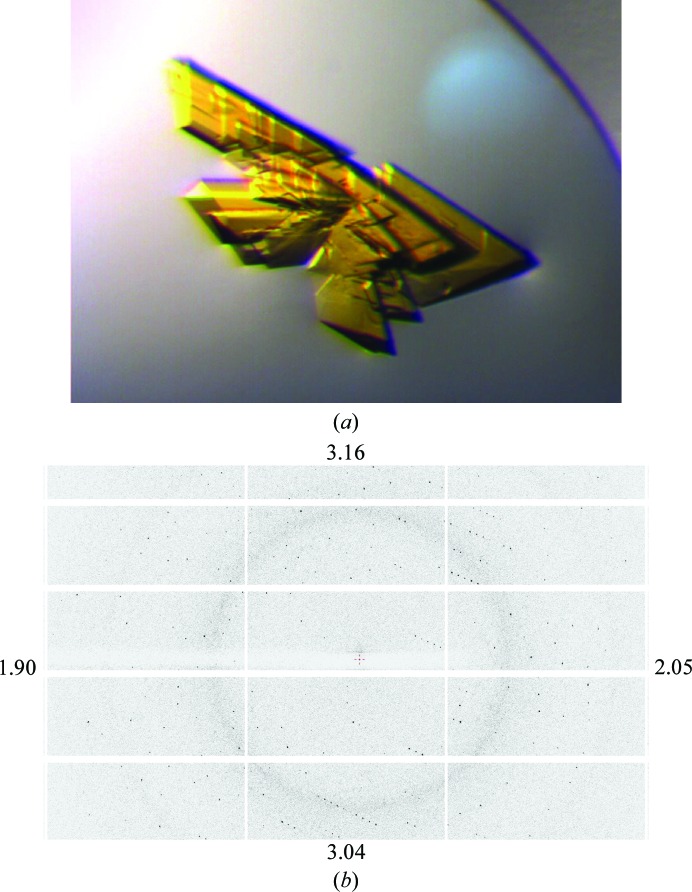
(*a*) Example of a multiple crystal of the SIP similar to that used for data collection on beamline I04 at DLS. (*b*) Detailed view of a SIP diffraction image obtained using a Pilatus 6M-F detector on beamline I04 at DLS. The numbers at the edge indicate the corresponding resolution limits. In the faintly visible diffuse scattering ring at ∼3.8 Å resolution, the maximum number of counts per pixel away from Bragg reflections is 3.

The crystal which was used for data collection was multiple, similar to that shown in Fig. 2 ▸. However, it was possible to obtain good-quality diffraction data from one of the single-crystal components, and no special precautions were needed in processing despite the apparent split crystal. No colour change of the crystal was observed during data collection, indicating an unchanged redox state of the FAD throughout X-ray exposure.

The 22% PEG 3350 concentration was sufficient to provide adequate crystal cryoprotection. In the partial diffraction image shown in Fig. 2 ▸, the lack of ice rings can be appreciated; although a weak diffuse scattering ring from the solvent is present, the maximum number of counts per pixel is not greater than 3.

**Figure 3:**
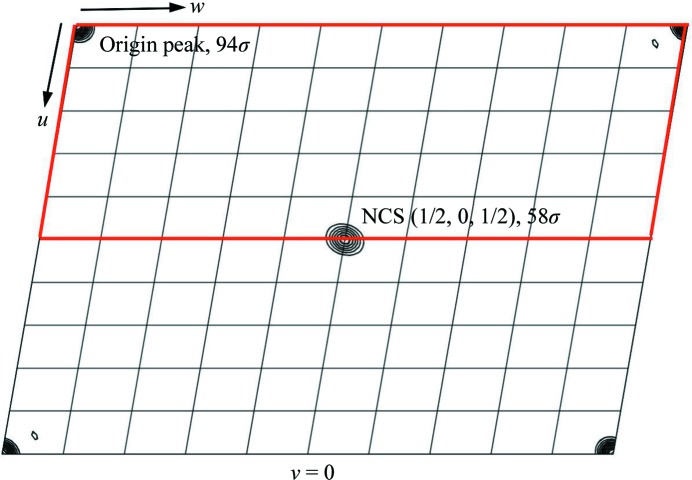
*v* = 0 section of a native Patterson map calculated using data to 2.5 Å resolution showing the presence of a strong non-origin peak at coordinates (1/2, 0, 1/2) consistent with pseudo-*B*-centring. Contour levels are drawn every 10 map r.m.s.d. units between 5 and 100 r.m.s.d. units. The section is drawn between 0 ≤ *u* ≤ 1 and 0 ≤ *w* ≤ 1 and the asymmetric unit is outlined in red.

The SIP crystals belonged to the monoclinic space group *P*2_1_, with unit-cell parameters *a* = 48.04, *b* = 78.31, *c* = 67.71 Å, β = 99.94°. Cell-volume considerations (Matthews, 1968 ▸; Kantardjieff & Rupp, 2003 ▸) indicate that there are two SIP monomers in the asymmetric unit, with a *V*_M_ of 2.13 Å^3^ Da^−1^ and an estimated solvent content of 42.4%. Although a self-rotation plot failed to reveal any significant trace of non­crystallographic twofold rotation axes, a native Patterson map calculated using data to 2.5 Å resolution displayed a strong non-origin peak at coordinates (1/2, 0, 1/2) with a peak height 61% of that of the origin, suggesting translational noncrystallographic symmetry consistent with a pseudo-*B*-centred lattice (Fig. 3 ▸). The overall twinning score was 2.28; thus, the data do not appear to be twinned.

Attempts will be made to determine the crystal structure by molecular replacement (MR) using the previously determined SIP structures PDB entries 2gpj (2.2 Å resolution) and 4yhb (1.89 Å resolution), which show 32 and 28% sequence identity, respectively, as templates. The higher resolution crystal structure of the SIP from *S. frigidimarina* will provide crucial information regarding the molecular mechanism underlying iron acquisition in microorganisms.

## Figures and Tables

**Table 1:** Macromolecule-production information

Source organism	*S. frigidimarina* NCIMB 400
DNA source	*S. frigidimarina* NCIMB 400
Forward primer (5′–3′)	ATG AAT AAC CAA TCA GCT AAA AAA TCT CC
Reverse primer (5′–3′)	CTA CAA CGG CTG CAT CTG CTT TTG
Cloning vector	pETBlue-1 (Novagen)
Expression vector	pETBlue-1 (Novagen)
Expression host	*E. coli* Tuner (DE3) pLacI
Complete amino-acid sequence of the construct produced	MNNQSAKKSPTRLTYISDIIEISPYLRRLVLSGEQLANFPADQQGAYVKVLIPQPGETTVNMTLTGPNAAIKRSYTIREFDPVRGQLSLDFVINKHTGPATDWAKLANVGDTVAIAGPGPLKMNRFDFNDYLLFGDSTSINAVDALIKRLPATAKGHIIMLVNSHQEQALLSQHPLLKTHWLVLNDSITAEQQIDWLLDKLELFGDLPAVTQVFVGLEATQVRVIKQYLLEQQQLPLSSISATGYWKRNTDADTFGKQKQMQPL
Theoretical pI†	7.6

† Calculated using *Protein Calculator* (http://protcalc.sourceforge.net/).

**Table 2:** Crystallization conditions

Method	Hanging drop
Plate type	EasyXtal 15-Well Tool (Qiagen)
Temperature (°C)	18
Protein concentration (mg ml^−1^)	10
Buffer composition of protein solution	20 m*M* Tris–HCl pH 7.6, 50 m*M* NaCl
Composition of reservoir solution	22% PEG 3350, 0.22 *M* magnesium formate dihydrate
Volume and ratio of drop	2 µl, 1:1
Volume of reservoir (µl)	500

**Table 3:** Data collection and processing

Diffraction source	Beamline I04, DLS
Wavelength (Å)	0.9795
Temperature (K)	100
Detector	Pilatus 6M-F
Rotation range per image (°)	0.10
Total rotation range (°)	180
Exposure time per image (s)	0.040
Space group	*P*2_1_
*a*, *b*, *c* (Å)	48.04, 78.31, 67.71
α, β, γ (°)	90, 99.94, 90
ISa	12.0
Resolution range (Å)	18.6–1.35 (1.39–1.35)
Total No. of reflections	357812 (26206)
No. of unique reflections	107545 (7947)
Completeness (%)	99.4 (99.6)
Multiplicity	3.3 (3.3)
〈*I*/σ(*I*)〉†	7.1 (1.3)
*R*_meas_	0.098 (0.970)
Overall *B* factor from Wilson plot (Å^2^)	15.2

† Mean *I*/σ(*I*) < 2.0 at 1.45 Å.

## References

[bb1] Blommel, P. G., Becker, K. J., Duvnjak, P. & Fox, B. G. (2007). *Biotechnol. Prog.* **23**, 585–598. 10.1021/bp070011x PMC2747370 17506520

[bb2] Brickman, T. J. & McIntosh, M. A. (1992). *J. Biol. Chem.* **267**, 12350–12355. 1534808

[bb3] Butterton, J. R. & Calderwood, S. B. (1994). *J. Bacteriol.* **176**, 5631–5638. 10.1128/jb.176.18.5631-5638.1994 PMC196765 8083157

[bb5] Crosa, J. H. & Walsh, C. T. (2002). *Microbiol. Mol. Biol. Rev.* **66**, 223–249. 10.1128/MMBR.66.2.223-249.2002 PMC120789 12040125

[bb6] Deneer, H. G., Healey, V. & Boychuk, I. (1995). *Microbiology*, **141**, 1985–1992. 10.1099/13500872-141-8-1985 7551061

[bb7] Holland, H. D. (2006). *Philos. Trans. R. Soc. Lond. B Biol. Sci.* **361**, 903–915. 10.1098/rstb.2006.1838 PMC1578726 16754606

[bb8] Kabsch, W. (2010). *Acta Cryst.* D**66**, 125–132. 10.1107/S0907444909047337 PMC2815665 20124692

[bb9] Kantardjieff, K. A. & Rupp, B. (2003). *Protein Sci.* **12**, 1865–1871. 10.1110/ps.0350503 PMC2323984 12930986

[bb10] Li, K., Chen, W.-H. & Bruner, S. D. (2015). *Biochemistry*, **54**, 3989–4000. 10.1021/acs.biochem.5b00354 26043104

[bb11] Matthews, B. W. (1968). *J. Mol. Biol.* **33**, 491–497. 10.1016/0022-2836(68)90205-2 5700707

[bb12] Miethke, M., Hou, J. & Marahiel, M. A. (2011). *Biochemistry*, **50**, 10951–10964. 10.1021/bi201517h 22098718

[bb13] Miethke, M. & Marahiel, M. A. (2007). *Microbiol. Mol. Biol. Rev.* **71**, 413–451. 10.1128/MMBR.00012-07 PMC2168645 17804665

[bb14] Neilands, J. B. (1981). *Annu. Rev. Biochem.* **50**, 715–731. 10.1146/annurev.bi.50.070181.003435 6455965

[bb15] Ryndak, M. B., Wang, S., Smith, I. & Rodriguez, G. M. (2010). *J. Bacteriol.* **192**, 861–869. 10.1128/JB.00223-09 PMC2812465 19948799

[bb16] Saha, M., Sarkar, S., Sarkar, B., Sharma, B. K., Bhattacharjee, S. & Tribedi, P. (2016). *Environ. Sci. Pollut. Res. Int.* **23**, 3984–3999. 10.1007/s11356-015-4294-0 25758420

[bb17] Schalk, I. J. & Guillon, L. (2013). *Amino Acids*, **44**, 1267–1277. 10.1007/s00726-013-1468-2 23443998

[bb18] Studier, F. W. (2005). *Protein Expr. Purif.* **41**, 207–234. 10.1016/j.pep.2005.01.016 15915565

[bb19] Wandersman, C. & Delepelaire, P. (2004). *Annu. Rev. Microbiol.* **58**, 611–647. 10.1146/annurev.micro.58.030603.123811 15487950

[bb4] Winn, M. D. *et al.* (2011). *Acta Cryst.* D**67**, 235–242.

[bb20] Winter, G. (2010). *J. Appl. Cryst.* **43**, 186–190.

